# Higher Levels of Pro-inflammatory Cytokines Are Associated With Higher Levels of Glutamate in the Anterior Cingulate Cortex in Depressed Adolescents

**DOI:** 10.3389/fpsyt.2021.642976

**Published:** 2021-04-15

**Authors:** Tiffany C. Ho, Giana I. Teresi, Jillian R. Segarra, Amar Ojha, Johanna C. Walker, Meng Gu, Daniel M. Spielman, Matthew D. Sacchet, Fei Jiang, Yael Rosenberg-Hasson, Holden Maecker, Ian H. Gotlib

**Affiliations:** ^1^Department of Psychiatry and Weill Institute for Neurosciences, University of California, San Francisco, San Francisco, CA, United States; ^2^Department of Psychology, Stanford University, Stanford, CA, United States; ^3^Center for Neuroscience, University of Pittsburgh, Pittsburgh, PA, United States; ^4^Department of Radiology, Stanford University, Stanford, CA, United States; ^5^Center for Depression, Anxiety, and Stress Research, McLean Hospital and Harvard Medical School, Belmont, MA, United States; ^6^Department of Epidemiology and Biostatistics, University of California, San Francisco, San Francisco, CA, United States; ^7^Department of Microbiology and Immunology, Stanford University, Stanford, CA, United States

**Keywords:** glutamate, depression, magnetic resonance spectroscopy, interleukin-6, ascorbate

## Abstract

Animal models of stress and related conditions, including depression, have shown that elevated peripheral levels of inflammatory cytokines have downstream consequences on glutamate (Glu) in the brain. Although studies in human adults with depression have reported evidence of higher inflammation but lower Glu in the anterior cingulate cortex (ACC), the extent to which peripheral inflammation contributes to glutamatergic abnormalities in adolescents with depression is not well-understood. It is also unclear whether antioxidants, such as ascorbate (Asc), may buffer against the effects of inflammation on Glu metabolism. Fifty-five depressed adolescents were recruited in the present cross-sectional study and provided blood samples, from which we assayed pro-inflammatory cytokines, and underwent a short-TE proton magnetic spectroscopy scan at 3T, from which we estimated Glu and Asc in the dorsal ACC. In the 31 adolescents with usable cytokine and Glu data, we found that IL-6 was significantly positively associated with dorsal ACC Glu (β = 0.466 ± 0.199, *p* = 0.029). Of the 16 participants who had usable Asc data, we found that at higher levels of dorsal ACC Asc, there was a negative association between IL-6 and Glu (interaction effect: β = −0.906 ± 0.433, *p* = 0.034). Importantly, these results remained significant when controlling for age, gender, percentage of gray matter in the dorsal ACC voxel, BMI, and medication (antidepressant and anti-inflammatory) usage. While preliminary, our results underscore the importance of examining both immune and neural contributors to depression and highlight the potential role of anti-inflammatory compounds in mitigating the adverse effects of inflammation (e.g., glutamatergic neuroexcitotoxicity). Future studies that experimentally manipulate levels of inflammation, and of ascorbate, and that characterize these effects on cortical glutamate concentrations and subsequent behavior in animals and in humans are needed.

## Introduction

Depression is an impairing and prevalent disorder that commonly emerges during adolescence (1, 2). While there is growing evidence that elevated inflammation, measured by peripheral levels of pro-inflammatory cytokines and proteins, are implicated in the development and maintenance of depression in adults [(3, 4); for reviews see also (5, 6)], the role of these immune markers in adolescent depression is unclear [although see (7, 8) for recent research in non-depressed adolescents]. It is well recognized both that life stress is one of the most potent risk factors for depression during adolescence (9–11), and that the effects of stress on the immune and nervous systems are critical in understanding the onset and persistence of depression. Extensive research has shown that psychosocial stressors activate the immune system by initiating a cascade of inflammatory responses, including increased production of pro-inflammatory cytokines in the peripheral nervous system (12). Importantly, peripheral cytokines can cross and alter the permeability of the blood-brain barrier (13) and influence processes that affect glutamate metabolism through a variety of mechanisms, including, but not limited to, increased production of quinolinic acid-which binds to glutamatergic NMDAreceptors and provokes release of glutamate into the synapse-and the impediment of astrocyte reuptake of glutamate (5, 12, 14, 15). Indeed, preclinical data indicate that inflammatory cytokines stimulate glutamate release that, over time, leads to cell apoptosis and to damage to oligodendrocytes (16, 17). Thus, glutamatergic excitotoxicity may be one mechanism by which inflammatory cytokines effect depression-related changes both in the brain and in behavior (18).

Consistent with this framework, investigators have conducted studies in adults of translocator protein 18 kDA (TSPO)-positron emission topography (PET) binding that index microglial activation by measuring TSPO expression; these researchers report higher TSPO binding in the ACC in patients with depression compared to healthy controls (19, 20). Neuroinflammation and mediators of the immune response (e.g., microglia) in the central nervous system cannot be measured non-invasively; however, technologies such as proton magnetic resonance spectroscopy (MRS) can be used to measure non-invasively the downstream effects of peripheral cytokines on levels of neurotransmitters, including glutamate. Studies using MRS to examine depression-related alterations in glutamate have primarily focused on the anterior cingulate cortex [ACC; for reviews and meta-analyses, see (21, 22)], a large brain region with distinct subdivisions (23). While these recent reviews report that the published literature thus far has identified *lower* glutamate in the ACC in depressed individuals compared to healthy controls, these patterns have been equivocal in the nine MRS studies conducted with depressed adolescents. Prior studies have focused primarily on adults with depression, in which measures of glutamate are often confounded by medication usage, chronicity of illness, and age-related effects on overall brain development (e.g., thinner gray matter) that likely complicate the nature of how, and the extent to which, inflammation affects glutamate in the context of MDD. Importantly, with the exception of one study (24), all MRS studies of depressed adolescents conducted to date have each recruited fewer than 17 depressed adolescents (22), and many have acquired spectral data on MR scanners at 1.5 Tesla (22), which have poorer signal-to-noise ratios and more limited resolution for resolving spectral resonances than do scanners at 3 Tesla.

The present study was designed to examine the role of glutamatergic abnormalities in adolescent depression by testing associations between peripheral levels of inflammation, indexed by pro-inflammatory cytokines, and levels of Glu in the dorsal ACC (dACC) in clinically depressed adolescents scanned at 3 Tesla. Our focus on the dACC is motivated in part by previous studies that have identified functional and structural abnormalities in this division of the ACC in depressed vs. non-depressed adolescents. From a neurodevelopmental perspective, the dACC has been shown to be a key region in which there are dramatic changes in connectivity with limbic and cortical regions that underlie affective processing and cognitive control during adolescence (25, 26). Thus, it is likely that insults in the form of psychosocial stress and psychiatric disease have a profound impact on dACC development and, subsequently, on psychosocial skills associated with adaptive emotion regulation (23, 27). Indeed, our group has reported that depressed adolescents exhibit lower dACC network connectivity than do psychiatrically healthy controls and, further, that dACC network connectivity is associated with an earlier age of depression onset (28). Together, these results suggest that the dACC is a critically important region to examine in understanding the neurobiological substrates of adolescent depression.

An exploratory aim of the study was to examine the role of antioxidants in a model of inflammatory contributions to glutamatergic metabolism in adolescent depression. Basic researchers have begun to report that antioxidants, such as ascorbate (Asc, also known as Vitamin C), buffer against the neurotoxic effects of excessive glutamate in neurons, and may serve a neuroprotective role against glutamatergic excitotoxicity (29–32). These findings, however, have yet to be replicated in humans.

To address these gaps, we recruited 55 clinically depressed adolescents who underwent short-TE MRS scans that permitted modeling of Glu and Asc resonances at 3 Tesla, and who provided blood samples from which we assayed inflammatory cytokines. We tested whether peripheral levels of inflammation were positively associated with glutamate concentrations in the dorsal ACC, and explored whether levels of ascorbate in the dACC moderated the associations between peripheral levels of inflammation and dACC glutamate.

## Methods and Materials

### Participants

Fifty-five adolescents between the ages of 13 and 18 years were recruited from the San Francisco Bay Area community as part of a longitudinal study examining neurobiological mechanisms underlying adolescent stress and depression (18). We interviewed participants and their caregiver at an initial session to assess study eligibility (see below) using the Kiddie Schedule for Affective Disorders and Schizophrenia–Present and Lifetime [K-SADS-PL; (33, 34)], the Children's Depressive Rating Scale–Revised [CDRS-R; (35)], and the Family Interview for Genetics Studies [FIGS; (36)]. Eligible participants were invited to complete additional questionnaires, and to complete an MRI scan and provide blood samples at a subsequent session held ~2 weeks after the initial baseline session (interval: 10.85 ± 6.08 days). Inclusion criteria for potentially depressed adolescents included being 13–18 years of age, being fluent in English, and having a depressive disorder (Major Depressive Disorder, Dysthymia, or Depressive Disorder Not Otherwise Specified) based on a combination of the K-SADS-PL and CDRS-R (i.e., *t*-scores ≥55 or raw scores ≥30) for those who did not meet full criteria for MDD or Dysthymia in the K-SADS-PL screening, provided that the participant also endorsed at least 2 symptoms of MDD or Dysthymia in the K-SADS-PL screening [see (18) for more details]. Exclusion criteria included meeting lifetime or current criteria for Mania, Psychosis, or Alcohol Dependence (based on DSM-IV) or Moderate Substance Use Disorder with substance-specific threshold for withdrawal (based on DSM-V), premenarchal status (for females), history of concussion within the past 6 weeks or history of any concussion with loss of consciousness, contraindications for MRI scanning (e.g., braces, metal implants, or claustrophobia), and any serious neurological or intellectual disorders that could interfere with the ability to complete study components. The study was approved by the Institutional Review Boards (IRBs) at Stanford University and the University of California, San Francisco. All participants and their legal guardian(s) gave written assent and informed consent, respectively, in accordance with the Declaration of Helsinki, and were compensated for their participation.

### Depression Symptom Severity

Trained research assistants administered the CDRS-R to participants and their parents/legal guardians as a measure of depression symptom severity. The CDRS-R is a clinician-rated scale composed of 17 questions; both participants and caregivers were administered the first 14 questions to assess depressive symptomology, while the last 3 questions were rated based on the interviewer's observation of the adolescent participant to assess non-verbal characteristics of depression including depressed affect, listless speech, and hypoactivity. Interviewers integrated responses from both parent and child interviews to produce summary items ratings, which were then summed to attain a total summary score for each participant. All item ratings were discussed by a subset of the co-authors to ensure consistency across interviewers to maximize reliability and validity. The CDRS-R is the most widely used rating scale in clinical research trials for assessing the severity of depression and change in depressive symptoms in children and adolescents with depression. While there are no thresholds for distinguishing mild, moderate, and severe levels of depression beyond the cutoff score of 30 that is used to determine depression and the score of ≤28 that is used to determine remission, there is evidence that scores of 35–40 indicate mild depression (37). In our full sample, 37 participants (67.3%) met the clinical cutoff score of 40 on the CDRS-R, 15 participants (27.3%) scored 35–40, 2 participants (3.6%) scored 28–35, and 1 participant (1.8%) had a score <28. In our final analytic sample with usable ACC Glu and cytokine data, 20 participants (64.5%) had scores higher than 40 and 11 participants (35.5%) scored 35–40; no participants had scores <35.

### Pro-inflammatory Cytokines

Peripheral levels of pro-inflammatory cytokines were assayed from dried blood spot (DBS) samples. Blood samples were collected using mini contact-activated lancets (BD 366594 Microtainer, BD Biosciences, San Jose, CA) that were used to prick the participant's finger from their non-dominant hand after running their hand under hot water for 2 min. Blood spots were collected on 1.3 cm filter paper cards (Whatman #903, GE Healthcare, Piscataway, NJ) with ~150–250 μL amount of blood per spot; spots were then dried overnight at room temperature before being transferred to Ziplock bags with a desiccant for storage in a −20°C freezer. Sample extraction and Luminex analysis was performed by the Human Immune Monitoring Center at Stanford University. Samples were extracted and diluted 3-fold in the Luminex assay buffer prior to being run on a 62-plex Procarta plex assay (Thermo Fisher, Santa Clara, CA) on the Luminex FlexMap 3D. Custom Assay Chex control beads were added to all wells (Radix Biosolutions, Georgetown, Texas). Data were analyzed using MasterPlex software (Hitachi Software Engineering America Ltd., MiraiBio Group). Both median fluorescence intensity (MFI) and calculated concentration values (in pg/mL) were estimated for each analyte. Based on prior work demonstrating advantages of using MFI over concentration values for low abundant analytes from multiplex assays (38), we conducted all analyses using median fluorescence intensity (MFI) values (log-transformed). To correct for non-specific binding, we employed ordinary non-linear least squares (ONLS) regression by regressing cytokine values (log MFI) on CHEX4 values; the resulting residualized scores were thus used in subsequent statistical analyses (39). To minimize multiple comparisons, we focused our analyses on the pro-inflammatory cytokines IL-1β, IL-6, TNF-α, as these cytokines have been previously linked with depression (4, 7, 8, 40–43) and have been shown to be assayed reliably from DBS (44, 45). Of the 55 participants, 1 participant did not return after their initial assessment, and 1 did not feel comfortable providing a blood sample. Of the 53 participants who provided blood samples, 6 had samples that were collected and assayed differently (for the purposes of piloting protocols), and 9 did not provide sufficient blood volume for the assay.

### MR Scanning Acquisition

All MRI scans were acquired at the Stanford Center for Cognitive and Neurobiological Imaging (CNI) with a 3T GE Discovery MR750 (General Electric Healthcare, Milwaukee, WI, USA) and Nova 32-channel head coil (Nova Medical, Wilmington, MA, USA). Participants completed a T1-weighted anatomical scan based on the GE BRAVO IR-prep, fast spoiled gradient (SPGR) sequence: TR/TE/TI = 8.2/3.2/600 ms; flip angle = 12°; 156 axial slices; FOV = 256 mm; matrix = 256 mm × 256 mm, voxel resolution = 1 × 1 × 1 mm^3^; total scan time = 3 min 40 s. Participants also completed proton MRS scans based on a modification of the GE Healthcare PRESS product sequence, PROBE-p™. Two features were added to the product PROBE-p™ sequence for improved localization: (1) 16 step phase cycling (EXORCYCLE on the two refocusing RF pulses) and (2) application of a sensitive point echo planar (EP) waveform during acquisition to further eliminate out-of-slice artifact in the logical z direction (46–48). The bandwidth of the CHESS RF water-suppression pulses was reduced from 150 to 75 Hz to avoid suppression of the ascorbate peak at 4.1 ppm. We performed single voxel spectroscopy using this sequence with TE/TR = 35/2,000 ms, 128 averages, total scan time = 5 min 4 s on the graphically prescribed region of the dACC based on the 3D T1-weighted anatomical images using anatomical landmarks (all prescriptions confirmed by TCH).

### MR Spectral Processing

**Figure 1:**
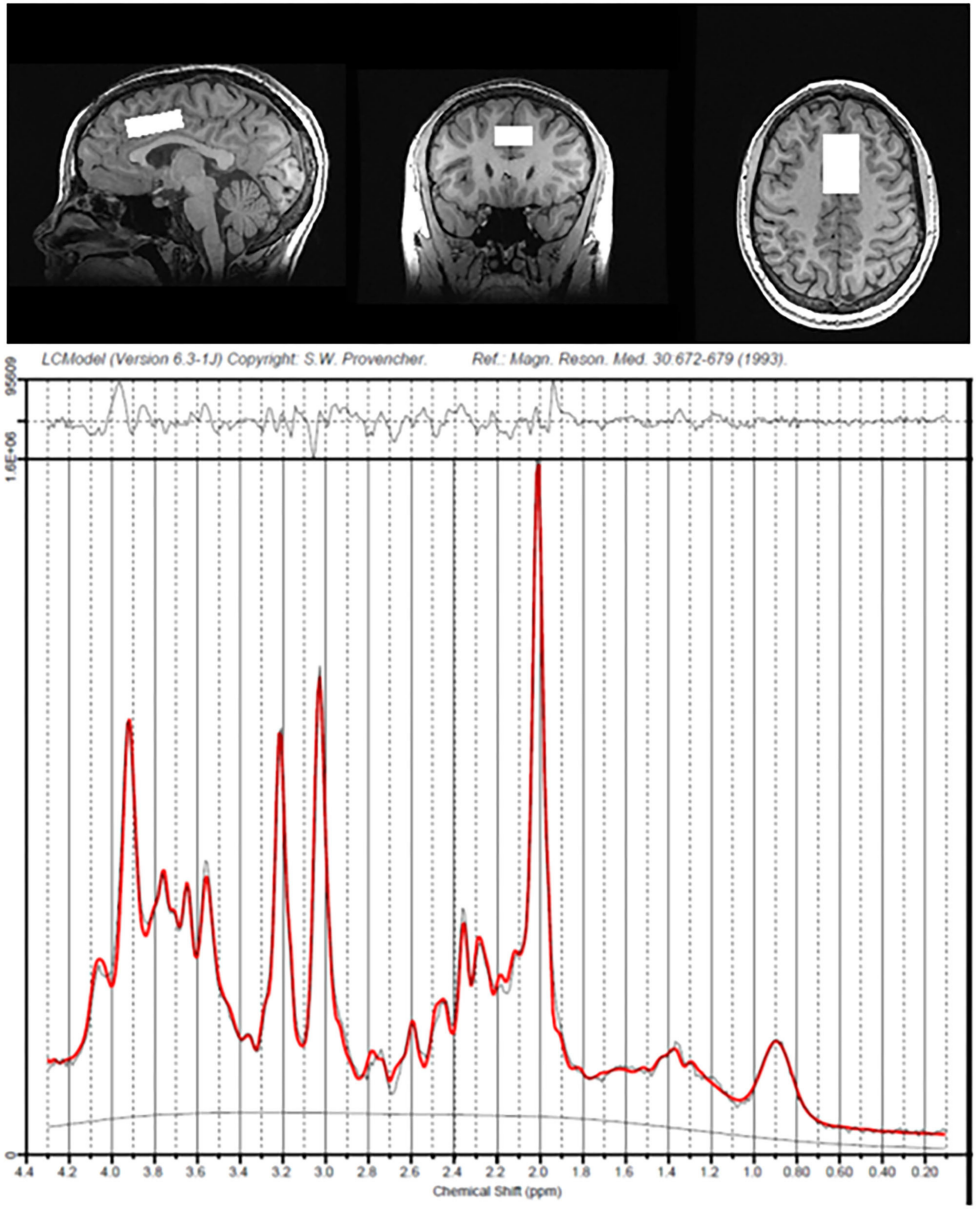
Voxel placement of dorsal anterior cingulate cortex and spectra data from a representative participant.

Concentrations of Glu, Asc, and other metabolites in the dACC were quantified and expressed as ratios to total creatine (i.e., creatine and phosphocreatine) levels using LCModel (49), which models *in vivo* spectrum as a linear combination of basis *in vitro* spectra from individual metabolites. Experimental Asc basis spectrum was acquired using the same sequence as for the *in vivo* studies from a custom built 50 mM Asc spherical phantom with pH of 7.2 at 37°C in an otherwise synthetic basis set to improve accuracy, due to the complexity of the Asc resonances and their dependence on temperature. Previous studies have indicated that Asc can be reliably measured from the human brain at 3 Tesla (50); independent data from our group also indicate that Asc at physiological concentrations can be reliably estimated from LCModel using this method at 3 Tesla with a mean Cramer-Rao Lower Bound (CRLB) of 16% in cortical gray matter (51). We used CRLB, a measure of the reliability of the fit, with a quality criterion set at ≤35% for each individual metabolite. All T1-weighted MR images were run through FreeSurfer 6.0 [(52); http://surfer.nmr.mgh.harvard.edu/fswiki/recon-all] to perform tissue segmentation and to estimate percentage of gray matter, white matter, and cerebrospinal fluid in the dACC voxel. For all statistical analyses involving Glu or Asc, we included percentage of gray matter in the prescribed dACC voxel as a covariate (see below). See Figure 1 for exemplary spectra from a representative subject. Of the 55 participants, 1 participant did not return after their initial assessment and 2 did not feel comfortable completing the MR scan. Of the 52 participants who completed the MRS scans, 44 provided usable data for Glu and 19 for Asc, based on CRLB criteria.

### Statistical Approach

All analyses were conducted in R (version 3.5.3; R Core Team). All associations between main variables of interest were tested using Pearson's correlations. We conducted multiple linear regressions to test whether baseline levels of pro-inflammatory cytokines were associated with concentrations of Glu in the dACC and whether levels of Asc in the dACC moderated any of these associations. To probe significant interaction effects, we applied the Johnson-Neyman procedure for continuous predictors using *sim_slopes* in R. In all statistical models, we covaried for age, gender (male, female, non-binary), psychotropic medication use (coded as a dichotomous variable), percentage of gray matter in the dACC voxel, Body Mass Index (BMI), and anti-inflammatory medication usage (coded as a dichotomous variable). We selected these covariates based on previous work identifying potential influences of these factors on both peripheral cytokine levels and MRS-based estimates of neurometabolites. To estimate standardized coefficient weights (β), all predictor and response variables were z-scored.

### Code Availability

Scripts for conducting functional clustering and statistical analyses can be found at: https://github.com/tiffanycheingho/TIGER/.

## Results

### Demographic and Clinical Characteristics

Demographic and clinical characteristics of participants at baseline are presented in Table 1. Of the 55 participants enrolled in the study, 11 did not provide usable Glu data, 36 did not provide usable Asc data, and 17 did not provide usable blood data. We compared the 16 participants who were not missing any data on any measure to the 39 participants who were missing data on at least one measure of interest on baseline demographic and clinical characteristics. None of the effect sizes of the differences were statistically significant (all *ps* > 0.081) and all were small or negligible, with the exception of highest level of parental education, which had a large effect size [Cramer's ϕ = 0.586, $\chi_{\left(5,\;50\right)}^{2}$ = 17.193, *p* < 0.01].

**Table 1:** Descriptive statistics of participant demographic and clinical characteristics.

**Variable**	**Descriptive statistics**
Age at V1 (years)	16.25 ± 1.32 (13.65 – 18.37)
Time between V1 and V2 (Days)	10.85 ± 6.08 (1 – 29) [1]
Sex (Female/Male)	65.45% (36)/34.55% (19)
**Gender**	
Male	34.55% (19)
Female	60.00% (33)
Non-binary/Other	5.45% (3)
**Sexual orientation^a^**	
Straight/Heterosexual	49.09% (27)
Gay/Lesbian	3.64% (2)
Bisexual	27.27% (15)
Other	5.45% (3)
Prefer not to say/Missing	14.55% (8)
Body Mass Index (BMI)	23.39 ± 5.63 (16.59 – 38.51) [2]
CDRS-R total	48.22 ± 12.12 (26 – 81)
Age of current depressive episode onset	13.65 ± 2.4 (4 – 17)
Number of depressive episodes	1.73 ± 1.04 (1 – 5)
**Current psychotropic medication**	**47.27% (26)**
Antidepressant	34.55% (19)
Antipsychotic	5.45% (3)
Stimulant	10.91% (6)
Benzodiazepine	1.82% (1)
Other^b^	12.73% (7)
Concurrent therapy	27.27% (15)
Therapy^c^	49.09% (27)
Anti-inflammatory medication^d^	12.73% (7)
**Ethnicity**	
Hispanic/Latinx	16.36% (9)
Not Hispanic/Latinx	83.64% (46)
**Race**	
White	47.27% (26)
Black/African American	3.64% (2)
American Indian/Alaska Native	3.64% (2)
Asian	18.18% (10)
Native Hawaiian/Pacific Islander	0% (0)
Multiracial	21.82% (12)
Other	5.45% (3)
**Highest parental education**	
Less than a high school diploma	0% (0)
High school graduate or equivalent (e.g., GED)	1.82% (1)
Some college (no degree)	9.09% (5)
Associate's degree (e.g., AA, AS)	3.64% (2)
Bachelor's degree (e.g., BA, BS)	27.27% (15)
Master's degree (e.g., MA, MS, MEd)	34.55% (19)
Doctoral or Professional degree (e.g., MD, DDS, DVM, Ph.D., EdD)	14.55% (8)
Unknown/Missing	9.09% (5)
**Annual household income**	
<$20,000	5.45% (3)
$20,000–$34,999	0% (0)
$35,000–$49,999	1.82% (1)
$50,000–$74,999	7.27% (4)
$75,000–$99,999	5.45% (3)
Over $100,000	69.09% (38)
Unknown/Missing	10.91% (6)
**Comorbidity**	**Lifetime reports**
Anxiety disorders^e^	43.64% (24)
Obsessive compulsive disorder	3.64% (2)
Eating disorders^f^	5.45% (3)
Disruptive, impulse control, and conduct disorders^g^	3.64% (2)
Post-traumatic stress disorder	20.00% (11)
Attention deficit hyperactivity disorder	23.64% (13)
Other^h^	1.82% (1)
Unknown/Missing	14.55% (8)

*All continuous values are reported as mean ± SD (min – max). Numbers in brackets [ ] indicate the number of missing or unusable responses. Categorical variables are reported as percentage (count). Lifetime comorbidities (includes past and current reports integrated across parent and child interviews) are reported as percentage (count). CDRS-R, Children's Depression Rating Scale-Revised*.

a *Other sexual orientations indicated by participants, with ( ) indicating count, include pansexual (1), queer (1), and demi-sexual (1)*.

b *Other medications taken by participants, with ( ) indicating count, include Gabapentin (1), Trazodone (4), Prazosin (1), Buspar (1), Dextromethorphan (1), and Cannabidiol (1)*.

c *Therapy indicates the percentage (count) of participants who reported attending therapy sessions for their depression in the 2 months prior to their first visit*.

d *Anti-inflammatory medication includes any anti-histaminergic, antibiotic, or steroid medications participants reported taking at the time of their session. In the subsample that provided a usable blood sample (n = 38), 7.89% (3) participants reported use of anti-inflammatory medication*.

e *Anxiety disorders reported with counts across participants: Panic Disorder (4), Social Phobia (11), Simple Phobia (5), Agoraphobia (2), Generalized Anxiety Disorder (16)*.

f *Eating Disorders: Anorexia Nervosa (2), Bulimia Nervosa (0), Eating Disorder Not Otherwise Specified (2)*.

g *Disruptive, Impulse Control, and Conduct Disorders: Oppositional Defiant Disorder (1), Conduct Disorder (1)*.

h *Other Disorders: Disruptive Mood Dysregulation Disorder (1), Austism Spectrum Disorder (1)*.

**Figure 2:**
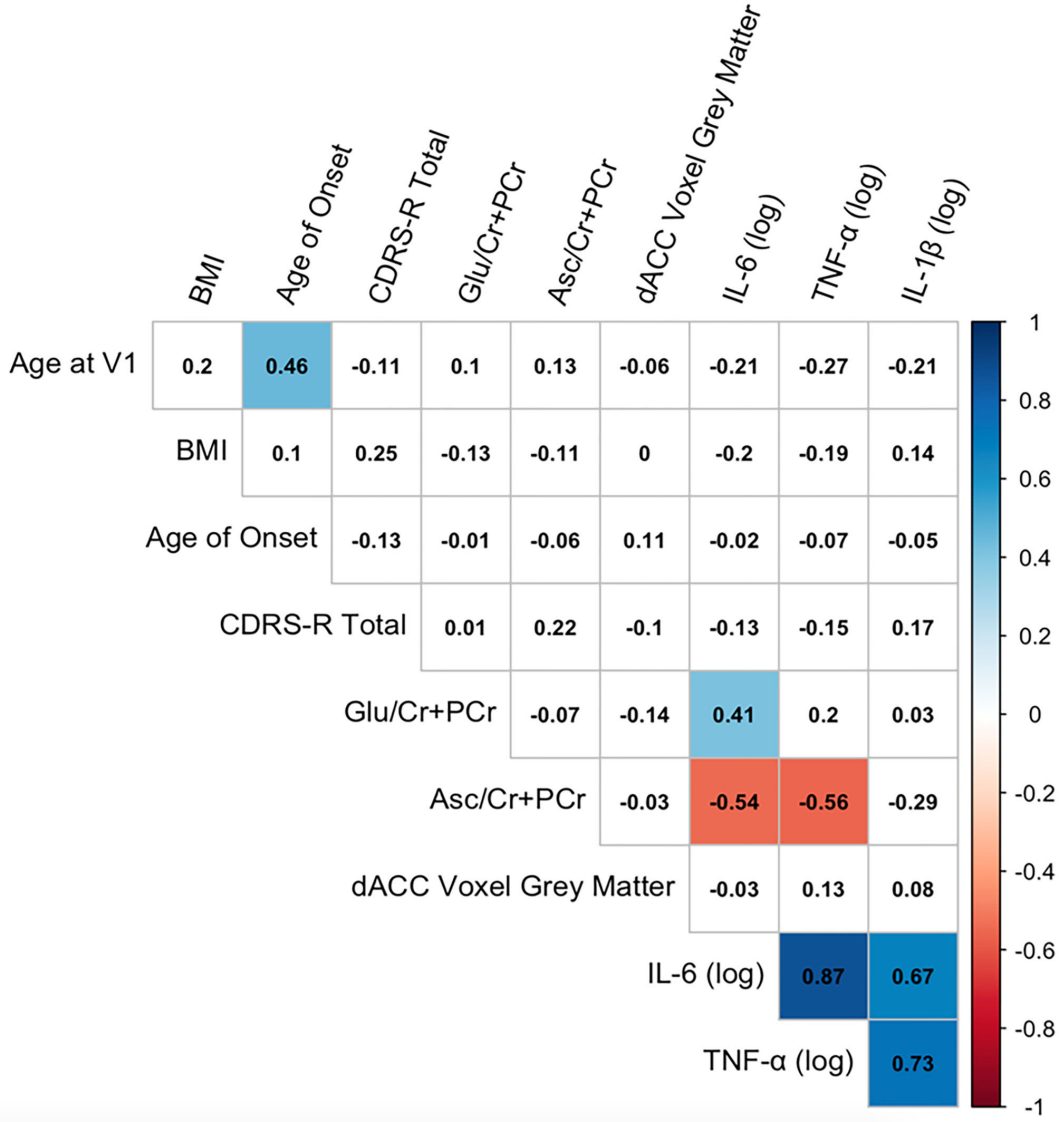
Correlation matrix among key variables of interest. Correlation matrix of primary predictor and outcome variables of interest, covariates, and clinical characteristics. Pearson correlation coefficients are presented in black text. Significant correlations at *p* < 0.05 (uncorrected) are highlighted in color (blue, positive correlations; red, negative correlations).

Descriptive statistics of the main variables of interest are presented in Table 2. A correlation matrix of the primary predictors and outcomes of interest, covariates, and clinical characteristics is presented in Figure 2. As expected, the inflammatory cytokines of interest were all highly intercorrelated (all *r*s > 0.67, all *p*s < 0.0001). IL-6 was positively correlated with Glu in the dACC (*r* = 0.41, *p* = 0.02), and both IL-6 and TNF-α were negatively correlated with Asc in the dACC (both *r*s < −0.54, both *p*s < 0.03). Finally, age of study assessment was associated with age of onset for current depressive episode (*r* = 0.46, *p* < 0.001).

**Table 2:** Descriptive statistics for main variables of interest.

**Variable**	**Descriptive statistics**
Glu/Cr + PCr	1.40 ± 0.17 (1.10 – 2.25) [11]
Asc/Cr + PCr	0.24 ± 0.05 (0.17 – 0.32) [36]
Gray Matter Percentage dACC Voxel (%)	0.56 ± 0.06 (0.35 – 0.68) [11]
IL-6 (log)	4.57 ± 0.13 (4.3 – 4.86) [17]
TNF-α (log)	4.83 ± 0.17 (4.51 – 5.13) [17]
IL-1β (log)	4.02 ± 0.17 (3.64 – 4.45) [17]

*All values are reported as mean ± SD (min – max). Numbers in brackets [ ] indicate the number of missing or unusable responses*.

### Higher Levels of IL-6 Are Associated With Higher Concentrations of Glutamate in the Dorsal Anterior Cingulate Cortex

**Figure 3:**
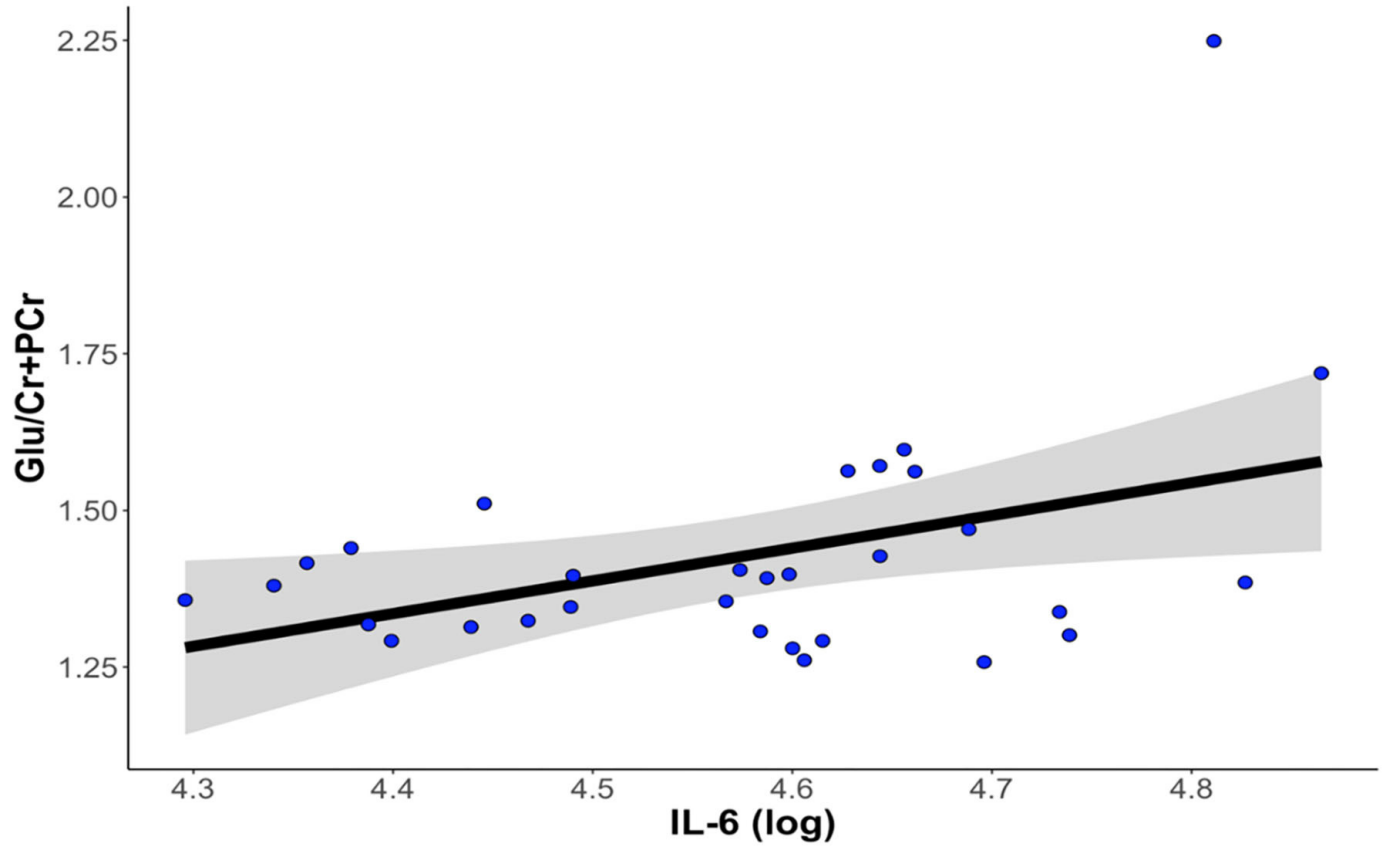
Higher levels of IL-6 at baseline are associated with higher levels of Glu in the dorsal anterior cingulate cortex. The correlation between IL-6 and dACC Glu remained statistically significant after controlling for age, gender, percentage of gray matter volume in the dACC voxel, antidepressant medication usage, and anti-inflammatory medication usage. dACC, dorsal anterior cingulate cortex; Glu, glutamate; IL-6, interleukin-6.

Thirty-one participants provided both usable cytokine and Glu data. Levels of IL-6 were significantly positively associated with concentrations of dACC Glu (β = 0.466 ± 0.199, *t*_22_ = 2.339, *p* = 0.029, ΔR^2^ = 0.199). Levels of IL-1β and TNF-α were not significantly associated with concentrations of Glu in the dorsal ACC (all *p*s > 0.288). See Figure 3 for more details.

One participant who exhibited abnormally high levels of Glu in the dACC was a statistical outlier despite having adequate CRLB for estimates of Glu. Although winsorizing the outlier data (based on a median-unbiased estimator interpolated from the sample without strict distribution assumptions, see *quantile* in R) did not change the significance of the association between IL-6 and dACC Glu (*p* < 0.05), removal of the outlier changed the statistical significance (*p* = 0.235). The associations between IL-1β and TNF-α with dACC Glu remained non-significant regardless of the treatment of the outlier (all *p*s > 0.397 when winsorizing, all *p*s > 0.764 when removing the statistical outlier).

### Exploring the Moderating Role of Ascorbate in the Dorsal Anterior Cingulate Cortex on the Association Between IL-6 and Glutamate in the Dorsal Anterior Cingulate Cortex

**Figure 4:**
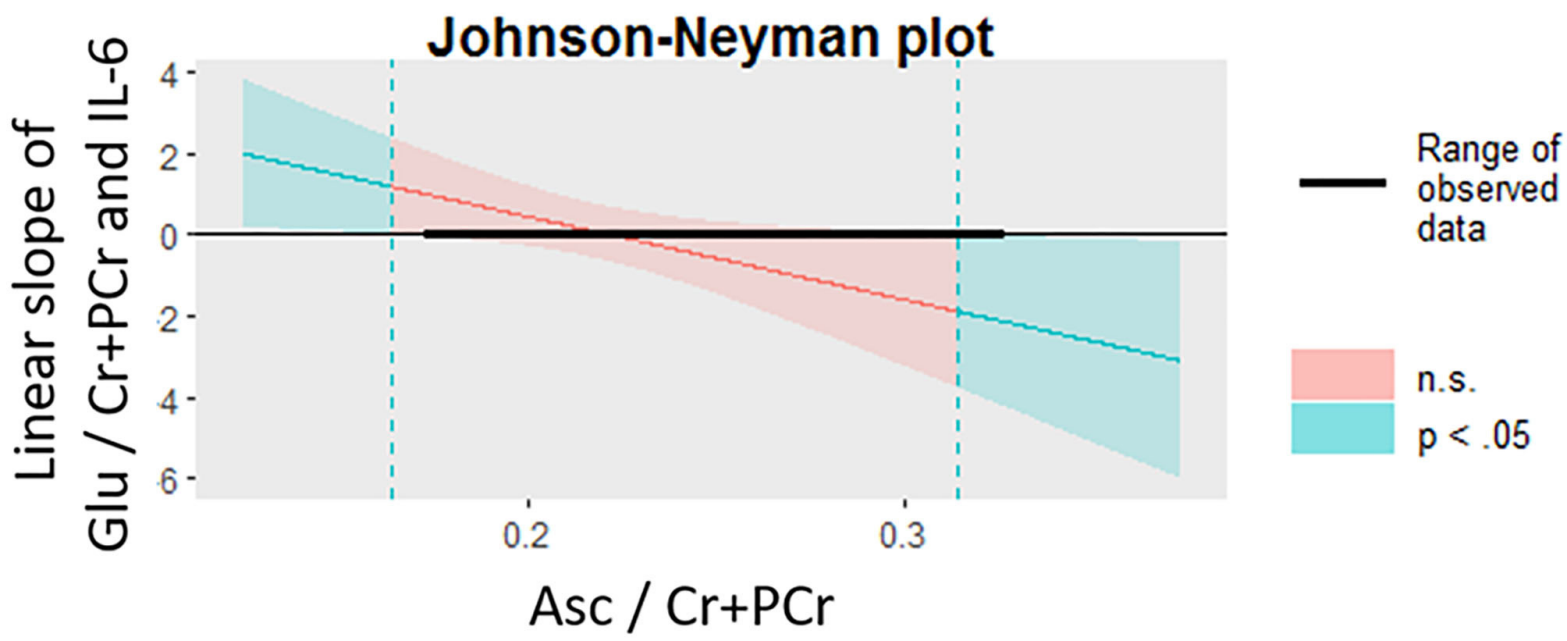
Levels of Asc in the dACC moderate the association between IL-6 and glutamate in the dACC. The Johnson-Neyman procedure was used to probe the interaction effect of dACC Asc and IL-6 on dACC Glu. At lower levels of Asc (<0.16, indicated by dotted teal line, left), the correlation between IL-6 and Glu was significantly positive (*p* < 0.05) whereas at higher levels of Asc (>0.32, indicated by dotted teal line, right), the correlation between IL-6 and Glu was significantly negative (*p* < 0.05). Asc, ascorbate; dACC, dorsal anterior cingulate cortex; Glu, glutamate; IL-6, interleukin-6.

Sixteen participants provided usable cytokine, Glu, and Asc data. Higher levels of Asc in the dorsal ACC moderated the associations between levels of IL-6 and Glu in the dorsal ACC, even after accounting for age, gender, BMI, percentage of gray matter in the voxel, and antidepressant and anti-inflammatory medication usage (interaction effect: β = −0.906 ± 0.433, *t*_6_ = −2.74, *p* = 0.034, ΔR^2^ = 0.557). We used the Johnson-Neyman procedure to compute the values of dACC Asc where the linear correlation between IL-6 and dACC Glu was statistically significant. We found that whereas Asc levels lower than 0.16 were associated with a significantly positive correlation between IL-6 and Glu, Asc levels higher than 0.32 were associated with a significantly negative correlation between IL-6 and Glu; Asc values between 0.16 and 0.32 were not associated with significant correlations between IL-6 and Glu. See Figure 4 for more details.

## Discussion

The present study was designed to examine, in depressed adolescents, associations between peripheral levels of inflammation, as indexed by pro-inflammatory cytokines, and concentrations of glutamate (Glu) in the dorsal anterior cingulate cortex (dACC), a region that has been implicated in adolescent depression in a range of structural and functional MRI studies but that has been relatively neglected in studies using MRS. We found that levels of IL-6, but not of IL-1β or TNF-α, were positively associated with concentrations of Glu in the dACC. In an exploratory analysis, we also found that higher concentrations of Asc in the dACC moderated the association between IL-6 and concentrations of Glu in the dACC, such that these markers were negatively correlated at higher levels of Asc only.

Our primary finding that higher levels of inflammatory cytokines, specifically IL-6, are associated with higher concentrations of glutamate in the dACC significantly advances our understanding of glutamatergic abnormalities in adolescent depression, particularly given the sparse and conflicting research in this area. First, our results are consistent with animal models demonstrating the role of inflammatory cytokines in contributing to glutamatergic excitotoxicity (12, 18); they appear to be inconsistent, however, with findings from MRS studies indicating lower concentrations of Glu in the ACC of depressed adults (22). One explanation for this discrepancy involves the specific region examined in this study. We focused on the dorsal division of the ACC based on findings of previous studies that abnormalities in this region may be a neurodevelopmentally sensitive marker of adolescent depression (28), in contrast to the rostral (pregenual) or medial ACC (22). Studies focused on the rostral and medial ACC in depressed adolescents, however, have found evidence of higher Glx (sum of glutamate and glutamine, a precursor to glutamate and GABA) in depressed adolescents with suicidal ideation than in healthy controls (53), as well as reductions in the ratio of glutamine to glutamate (Gln/Glu) in response to treatment-related symptom improvement (54). Although these studies examined Glx and Gln/Glu rather than solely Glu, their data are nonetheless consistent with our findings that higher levels of Glu may contribute to depressive symptomatology. Thus, a second explanation of the discrepancy between our findings and those of previous studies is that higher levels of Glu in the ACC may characterize depression that occurs at earlier developmental stages, when the consequences of chronically high levels of glutamate on brain structure (e.g., cell apoptosis, cortical thinning, etc.) have not yet had as large an impact. Future research with larger samples that include both adolescents and adults and that image the distinct portions of the ACC are needed to test these hypotheses more explicitly and to determine whether higher Glu in the ACC is an adolescent-specific indicator of depression.

Our finding that the positive association IL-6 and Glu in the dACC was found only at lower levels of Asc is broadly consistent with basic science work showing that Asc prevents excitotoxic damage to cells by inhibiting the binding of Glu to NMDA receptors and by limiting the effects of oxidative stress markers, including quercetin (29–32). Other studies have also shown that Asc is pharmacologically effective against glutamate-induced phosphorylation of AMPK, a mechanism of neuronal cell death (55). While preliminary, these results highlight the potential role of antioxidants in mitigating the downstream effects of inflammation on the brain, including glutamate metabolism. Indeed, several studies have reported that ascorbic acid supplements produce an antidepressant (and not anxiolytic, suggesting specificity) effect [for a review, see (56)]. The low toxicity and high tolerance of Asc may make Asc a possible adjuvant to first-line antidepressant treatments. An important direction of future research will be to test the extent to which anti-inflammatory strategies—including pharmacological agents, psychosocial therapies targeting stress reduction, or lifestyle and behavioral changes (e.g., changes in sleep, exercise, and/or diet)—affect levels of Asc in the brain and whether they help to reduce or prevent depression in adolescents.

Although the effect size of the association between IL-1β as well as TNF-α and Glu in the dACC was in the same direction as that of IL-6 and Glu, it is notable that we detected effects that were specific to IL-6. IL-1β, IL-6, and TNF-α are similar in their role in mediating immune responses; all three are released into circulation by innate immune cells to act on organs throughout the body [e.g., signaling the release of acute phase reactants such as C-reactive protein; (57)]. All three have also been shown to alter glutamate production (58, 59); in particular, TNF-α, has been strongly linked with glutamate-mediated oxidative stress and impairment of glutamate reuptake from astrocytes (59). Importantly, however, *in vivo* studies conducted with rodents have found that chronic stress paradigms that upregulate levels of IL-6 do so in the absence of increases in IL-1b and, further, that administration of IL-6 specifically produces depressive-like phenotypes in rodents (60). *In vivo* studies in human adolescents who are not clinically depressed have also showed that IL-6 predicts subsequent changes in depressive symptoms, although the extent of these effects appears to be conditional on other factors, including sex, stressful life events, and time or, possibly, developmental stage (7, 61). Our findings are consistent with much of the emerging human research in adults (12, 14, 15), by showing that in depressed adolescents higher levels of IL-6 specifically are associated with higher levels of glutamate. Nevertheless, it is critical that future studies with larger sample sizes replicate our findings and reconcile important differences between humans and animals in neuroimmune systems in order to facilitate forward and backward translation.

There are several limitations of our investigation that warrant additional discussion and that should be addressed in future research. First, our sample size was relatively small with a high degree of psychiatric comorbidity and medication use (including agents with some anti-inflammatory effects); it is important to note, however, that our study is one of the largest MRS studies to date conducted with depressed adolescents and the first to focus on the dACC (22), and also includes a sample that is representative of the type of patients who often present at clinics. Second, we did not exclude participants on the basis of medication history or usage. While we included both antidepressant and anti-inflammatory medications as statistical covariates in all of our analyses, it is possible that the effects of these medications nevertheless influenced our results. Bearing in mind issues concerning the generalizability of the findings (for example, many depressed adolescents seek treatment and are medicated), it will be important for future studies to consider recruiting unmedicated adolescents with depression. Third, we did not assess healthy control participants in our study. Future research will benefit from including psychiatrically healthy adolescents to clarify the extent to which the associations we observed are specific to adolescents who are depressed. Fourth, the reliability of Asc at 3 Tesla was significantly worse than the reliability of Glu; the advent of ultra-field MR imaging (e.g., 7 Tesla) may prove to be especially fruitful in improving detection and quantification of Asc. Finally, our study was a naturalistic observational study, which precluded our ability to make causal interpretations of the associations among IL-6, Glu, Asc, and depressive symptoms. Studies are needed that experimentally manipulate levels of inflammation, and of ascorbate, and that characterize these effects on cortical glutamate concentrations and subsequent behavior in samples of both depressed and non-depressed adolescents.

Despite these limitations, this study is important in establishing the associations among peripheral levels of inflammation and concentrations of glutamate and ascorbate in the dACC in humans with depression. In a cohort of depressed adolescents, we found that higher levels of IL-6 were associated with higher concentrations of Glu in the dACC and, further, that higher concentrations of Asc in the dACC moderated this effect, such that the positive association between IL-6 and Glu in the dACC was present only at lower levels of Asc. Although preliminary, our results underscore the importance of examining both immune and neural contributors to depression in adolescence and highlight the potential role of anti-inflammatory compounds in mitigating the adverse effects of inflammation (e.g., glutamatergic neuroexcitotoxicity) in depressed adolescents.

## Data Availability Statement

The datasets used and analyzed during the current study will be made available by the corresponding author upon reasonable request.

## Ethics Statement

The studies involving human participants were reviewed and approved by Institution Review Board at Stanford University. Written informed consent to participate in this study was provided by the participants' legal guardian/next of kin.

## Author Contributions

TH designed the research. GT, JS, AO, and JW helped perform research. TH, GT, JS, AO, and YR-H analyzed the data. MG, DS, MS, FJ, YR-H, and HM provided additional analytic tools to assist with data analysis. TH and IG obtained funding support. All authors contributed to the writing of the manuscript.

## Conflict of Interest

The authors declare that the research was conducted in the absence of any commercial or financial relationships that could be construed as a potential conflict of interest.
